# Two Distinct Filopodia Populations at the Growth Cone Allow to Sense Nanotopographical Extracellular Matrix Cues to Guide Neurite Outgrowth

**DOI:** 10.1371/journal.pone.0015966

**Published:** 2010-12-30

**Authors:** Kyung-Jin Jang, Min Sung Kim, Daniel Feltrin, Noo Li Jeon, Kahp-Yang Suh, Olivier Pertz

**Affiliations:** 1 Interdisciplinary Program in Nano-Science and Technology, Seoul National University, Seoul, Republic of Korea; 2 School of Mechanical and Aerospace Engineering, Seoul National University, Seoul, Republic of Korea; 3 Department of Biomedicine, Institute for Biochemistry and Genetics, University of Basel, Basel, Switzerland; The University of Akron, United States of America

## Abstract

**Background:**

The process of neurite outgrowth is the initial step in producing the neuronal processes that wire the brain. Current models about neurite outgrowth have been derived from classic two-dimensional (2D) cell culture systems, which do not recapitulate the topographical cues that are present in the extracellular matrix (ECM) *in vivo*. Here, we explore how ECM nanotopography influences neurite outgrowth.

**Methodology/Principal Findings:**

We show that, when the ECM protein laminin is presented on a line pattern with nanometric size features, it leads to orientation of neurite outgrowth along the line pattern. This is also coupled with a robust increase in neurite length. The sensing mechanism that allows neurite orientation occurs through a highly stereotypical growth cone behavior involving two filopodia populations. Non-aligned filopodia on the distal part of the growth cone scan the pattern in a lateral back and forth motion and are highly unstable. Filopodia at the growth cone tip align with the line substrate, are stabilized by an F-actin rich cytoskeleton and enable steady neurite extension. This stabilization event most likely occurs by integration of signals emanating from non-aligned and aligned filopodia which sense different extent of adhesion surface on the line pattern. In contrast, on the 2D substrate only unstable filopodia are observed at the growth cone, leading to frequent neurite collapse events and less efficient outgrowth.

**Conclusions/Significance:**

We propose that a constant crosstalk between both filopodia populations allows stochastic sensing of nanotopographical ECM cues, leading to oriented and steady neurite outgrowth. Our work provides insight in how neuronal growth cones can sense geometric ECM cues. This has not been accessible previously using routine 2D culture systems.

## Introduction

Proper functioning of the nervous system requires functional connections between neurons. This requires undifferentiated neurons to extend neurites that will subsequently differentiate in axons and dendrites to wire the adult brain. Studying the complex morphogenetic event of neurite outgrowth is not only important for understanding the development of the nervous system, but also tissue regeneration after nerve injury and the treatment of neuropathological conditions. Until now most of the work on neurite outgrowth at the cell biology level has been done using routine 2-dimensional (2D) culture systems. However, *in vivo*, cells interact with complex 3-dimensional anisotropic environments that display a different topology from the isotropic 2D environment. In this context, multiple ECM proteins are capable of forming large structures with different geometrical and size features ranging from tens of nanometers to micrometers. The highly organized structure of the ECM is essential for cell and tissue morphogenesis and remodeling [1]. By example, parallel bundles of collagen fibrils are found in connective tissues [2] and laminins assemble basement membrane structures [3]. Laminin tracks on the surface of Schwann cells are also important for neurite outgrowth and neuronal regeneration after a lesion [4]. In this case, laminin most likely assembles fibrillar structures [5]. Furthermore, in the developing nervous system, axons often follow ECM tracks that are oriented along structures such a blood vessels [6]. It is therefore reasonable to assume that precise topological features of the ECM are important for the cell's ability to interact with and perceive its environment. However, the importance of the ECM organization and topography at the micro- and nano-meter scale is still poorly understood. With recent technological advances in microfabrication, this now becomes accessible [7], [8]. Multiple reports document that the processes of cell migration or of the extension of neuronal processes from neurons are highly dependent on the geometrical topology of the surrounding ECM.

Neurons have been reported to respond to different ECM topologies in terms of morphology. When laminin is presented on aligned nanofiber scaffolds, neuronal processes can orient along these fibers compared to randomly oriented scaffolds [9]. Similar alignment behavior has also been observed when neurons are plated on micrometric laminin lines [10]. When human embryonic stem cells are plated on specific nanopatterns, they can effectively and rapidly differentiate into a neuronal lineage without the use of differentiation-inducing agents [11], [12], [13]. Thus, ECM nanoscale topography not only regulates cell morphology but also cell fate. While the combination of such nanotopographic cues with biochemical cues such as retinoic acid further enhances neuronal differentiation, nanotopography showed a stronger effect compared to retinoic acid alone on an unpatterned surface [13]. The mechanisms by which nanotopographic ECM cues influence differentiation appear to involve changes in cytoskeletal organization and structure, potentially in response to the geometry and size of the underlying features of the ECM. This might influence the clustering of integrins in focal adhesions and the formation of actin stress fibers, and thus the adhesion and spreading of cells. Secondary effects, such as alterations in the effective stiffness perceived by the cell or differences in protein adsorption due to the structural features of the substrate are also possible [14]. However, the cellular mechanisms of cell fate control by ECM nanotopography remain largely unexplored.

One of the best characterized example of control of cell behavior by ECM topology has been observed during fibroblast cell migration [15]. It is well described that fibroblasts migrate about 1.5 times faster on ECM fibrils in 3D cell-derived matrices compared to the same ECM presented in a classic 2D environment. In this study, 1D micro-patterned ECM lines with precise size features (1–2 µm width) have been shown to recapitulate the cell migration behavior observed in cell-derived 3D ECM environments. This most likely occurs because these ECM lines are able to mimic the fibrillar nature of the ECM in a 3D environment. Importantly, such a pseudo 3D environment has provided a convenient platform to analyze cell migration using microscopy techniques that do not require confocality. This has given novel insight about the molecular mechanisms of how cells perceive and migrate in 3D versus 2D environments. Comparable results have also been observed during cell migration on similar patterns at the nanometer scale [16].

In this study, we sought to understand the molecular mechanisms of how neurons respond to matrix nanotopography during the process of neurite outgrowth. For that purpose, we explored in detail neuronal morphology and morphodynamics on nanopatterns. We find that when cells are challenged with a highly defined anisotropic, nanotopographic laminin substrate, distinct neurite outgrowth responses occur in comparison with the classic, isotropic 2D environment. Our data suggest that growth cone filopodia are the organelles that allow to sense these nanotopographic ECM cues to orient neurite outgrowth. Importantly, we find that oriented outgrowth is also coupled with steady neurite outgrowth. This allows for more robust neurite outgrowth on the nanotopographical versus the 2D ECM.

## Results

**Figure 1:**
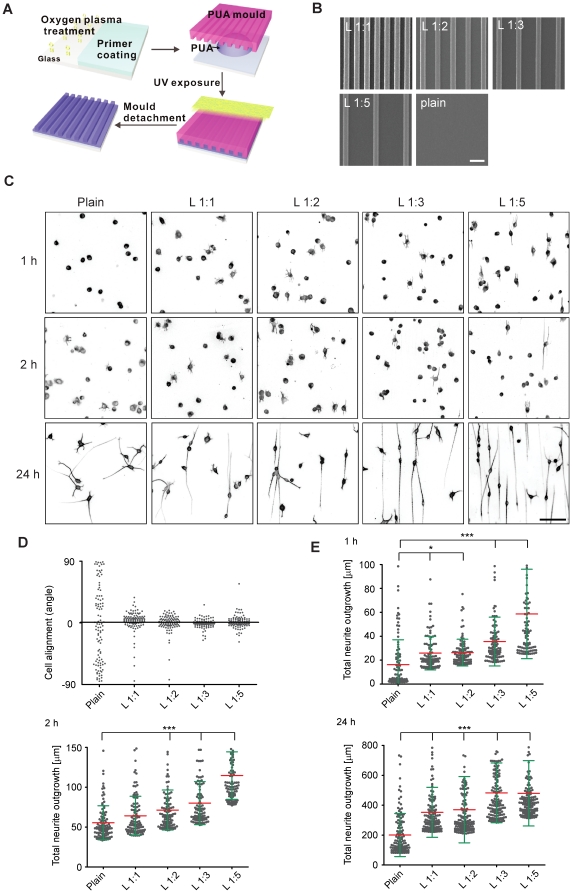
Neurite outgrowth response on nanotopographic pattern. (**A**) Schematics of nanopattern fabrication. (**B**) SEM images of PUA nanopattern with different ridge/groove ratios. (**C**) Representative images of tubulin-stained N1E-115 cells allowed to extend neurites on different plain and line patterns for 24 hours. (**D**) Quantification of neurite orientation at 24 hours. Angle measurements of neurite orientation compared to an arbitrary (plain substrate) or to the line direction (line substrate) are shown. Data is based on more than 100 cells for experiment. (**E**) Cumulative graph of the 20 percentile highest total neurite lengths on a per cell basis. Mean (red) and standard deviations (green) are shown. Data is based on 140 cells of 700 cells for each group. P values (Anova) between plain and line substrates <<0.0001 except 2 hours of line 1∶1 (no significance). Bars: (B) 1 µm; (C) 200 µm.

To explore how ECM nanotopology can regulate neurite outgrowth, we used ultraviolet-assisted capillary force lithography to construct ridge/groove pattern arrays on glass coverslips [17], [18]. Here, liquid polyurethane acrylate (PUA) is coated on a plasma-treated glass coverslip to which a PUA mold is applied (Fig. 1A). The cavities of this mold are filled by PUA through capillary force which is then cured by exposure to UV light. We fabricated different topographic patterns that were composed of arrays of parallel ridges that are 350 nm wide and 350 nm high, separated by grooves of 1, 2, 3, 5 times 350 nm width increments (1∶1, 1∶2, 1∶3, 1∶5 patterns). The fidelity with which we are able to produce such line patterns is illustrated by scanning electron micrographs (SEM) (Fig. 1B). We then used differentiated N1E-115 cells as a model system to compare the neurite outgrowth responses on classic 2D, laminin-coated coverslip (plain substrate) versus laminin that is presented on these line patterns (line substrate). Using fluorescently-labeled laminin, we found that this protein homogeneously coated the topographical patterns (Fig. S1A).

To evaluate the neurite outgrowth responses, we stained the microtubule cytoskeleton and the nuclei of the cells at different time points after plating and used automated image analysis to measure neurite length and orientation on the plain and line substrates (Fig. S1B). We observed that neurites align in the direction of the line pattern, whereas they extend randomly on the plain substrate (Fig. 1C and D). This orientation was not dependent on the spacing of the lines. Second, we found that the line pattern led to an increase in neurite length (Fig. 1C and E) which increases with groove width and peaks on the 1∶3 and 1∶5 patterns. As a control, we also evaluate a 1∶40 pattern, and found that neurite outgrowth was still oriented, was less robust than on the 1∶3 and 1∶5 patterns, but still more robust than on plain substrate (data not shown). Laminin coating of normal coverslips or coverslips that have been covered with a homogeneous PUA layer yielded similar results, showing that these different cell responses were not dependent on PUA (data not shown). Importantly, the size features of the ridges on the line substrate are smaller than a growth cone. Furthermore, we observed that the neurite is slightly deflected compared to the ridge direction. Orientation of neurite outgrowth does therefore not happen by physical trapping of the neurite in the grooves. Thus, the simple fact of altering the topographical state of which an ECM is presented to the cell drastically alters neurite orientation and outgrowth. Neurite orientation not only occurred with our neuronal-like neuroblastoma cell line, but similar results were also observed with freshly isolated primary cortical neurons that were plated on a 1∶5 line substrate coated with poly-L-ornithine and laminin (Fig. S2).

**Figure 2:**
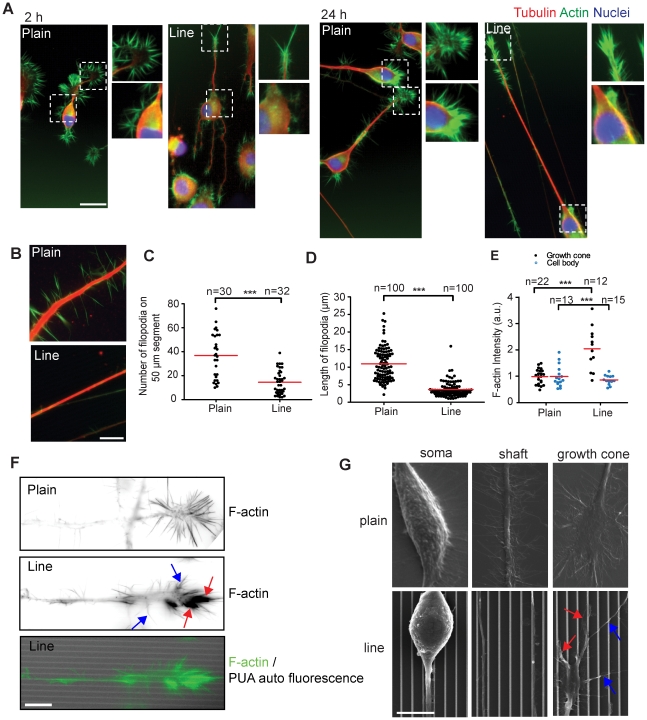
Neuronal morphology responses. (**A**) Fluorescence micrographs of N1E-115 cells immunostained for α-tubulin (red), F-actin (green), and nucleus (blue). Cells were allowed to extend neurites for 2 or 24 hours on the plain or line substrates. The magnified view of the white dotted box in each panel shows growth cone (right, top) and cell body (right, bottom). (**B**) Fluorescence micrographs of neurite shaft on plain and line substrates (Scale bar, 20 µm). Cells were immunostained as in (A). (**C**) Filopodia number on neurite shaft on plain versus line substrates. (**D**) Filopodia length on neurite shaft on plain versus line substrates. (**E**) Normalized F-actin intensity of growth cone and cell body on plain and line substrates. (**F**) High resolution pictures of neurite growth cones on plain and line substrates. Top two panels, F-actin phalloidin fluorescence micrographs of growth cones on plain and line substrates. Bottom panel, overlay of F-actin staining (green) and PUA autofluorescence (grey). (**G**) SEM images of N1E-115 cells plated on plain and line substrates. Black arrows represent robust filopodia latching on and aligned on the line pattern ridges. White arrows represent thin filopodia not aligned on the pattern ridges. (C), (D) and (E) P values (T-test) between plain and line <<0.0001. Means are shown in red. Bars: (A) 50 µm; (B) 20 µm; (G, F) 10 µm.

We next thought to understand the cellular mechanisms that allow the specific neuronal cell responses on the line substrate. For that purpose, we used the 1∶5 line substrate throughout this study since it leads to the most robust phenotype in terms of neurite length. We first immunostained the cells on plain and 1∶5 line substrates to visualize the F-actin and tubulin cytoskeletons 2 and 24 hours after plating (Fig. 2A). Surprisingly, we found that a higher amount of filopodia was typically observed on the soma, neurite shaft and growth cone of cells on plain versus line substrate. Quantitation revealed a two fold increase of filopodia number on the neurite shaft on plain versus line substrate (Fig. 2B and C). These filopodia were also longer (Fig. 2B and D). While growth cones were highly spread and displayed a high density of randomly oriented filopodia on plain substrate, less spread, streamlined growth cones with fewer filopodia occurred on line substrate. These growth cones exhibited thick filopodia that aligned in the direction of the pattern ridges and displayed a high F-actin content as observed by phalloidin staining (Fig. 2A and E). This was especially evident with high resolution images of growth cones on the line substrate, and, in addition to the thick, F-actin rich aligned filopodia revealed a second population of thin, F-actin poor filopodia that were not aligned with the lines (Fig. 2F). Similar results were also observed in SEM experiments and revealed that thick filopodia align and intimately adhere along the top of the line ridges (Fig. 2G, red arrows), whereas thin, unaligned filopodia only interact with the line ridges at discrete points (Fig. 2G, black arrows).

**Figure 3:**
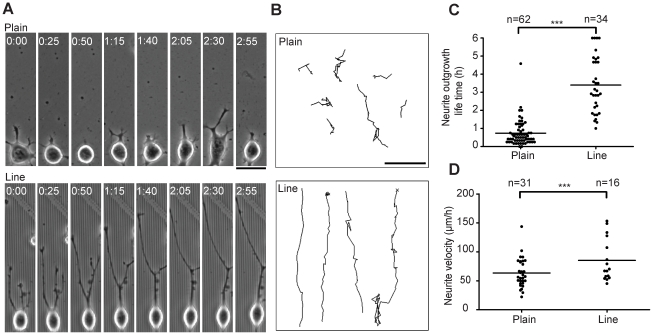
Neurite morphodynamics. (**A**) Phase contrast timelapse series of cells on plain and line substrates. (**B**) Representative neurite tip tracks on plain and line substrates. (**C**) Quantification of neurite outgrowth life time. (**D**) Quantification of neurite velocity on plain and line substrates. P values (T-test) <<0.0001 in both (C) and (D). Bars: (A,B) 25 µm.

We then used phase contrast time-lapse microscopy to study the morphodynamics of neurite outgrowth on plain and line substrates. We observed that neurites exhibited a highly unstable behavior that consisted of multiple cycles of neurite protrusion and retraction events on the plain substrate (Fig. 3A, Movies S1 and S2). In the early phases of the process, this often resulted in re-absorption of the neurite by the cell soma which was followed by the creation of a new initiation site and the outgrowth of a new neurite. In contrast, on the line pattern, neurites almost never retracted and thus outgrowth was steady (Fig. 3A). We tracked neurite tip trajectories and found that neurite outgrowth on plain substrate typically occurred for a period of 30 min before a retraction event occurred (Fig. 3B and C). This neurite extension lifetime was extended to 180 minutes on the line substrate with retraction events typically occurring at neurite branch points (Fig. 3C). This allowed for the elimination of the branch points and led the cell to adopt two unbranched neuronal processes that align in the direction of the line pattern. We found that neurite tip velocity was only modestly increased on the line versus plain substrate (Fig. 3D). Soma motility was also affected. On plain substrate, the soma displayed a highly motile behavior (average speed  = 60 µm/hour) consisting of random bursts of migratory behavior. On the line substrate, cells were much less motile (average speed  = 20 µm/hour) (Fig. S3, Movie S2). Thus, the line substrate not only allows neurite orientation, but also switches off the dynamic unstable behavior of neurites and the motile behavior of cells observed on plain substrate.

**Figure 4:**
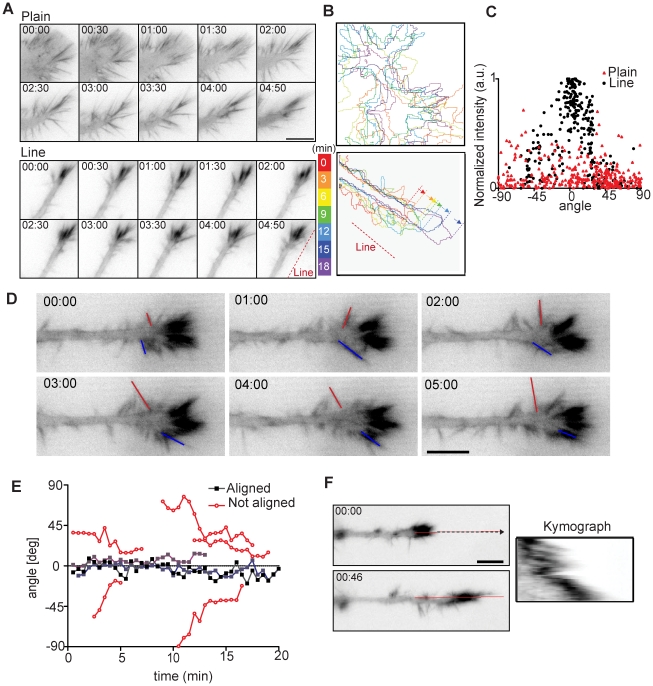
Growth cone filopodial dynamics. (**A**) Time-lapse series of growth cone filopodial dynamics on plain and line substrates. Cells were transfected with Lifeact-GFP and imaged using fluorescence microscopy. Time is in minutes:seconds. (**B**) Cellular outlines were captured and overlaid to show movement of neurite tip. (**C**) Analysis of correlation of filopodia alignment (angle compared to line direction) and normalized F-actin intensity on plain and line substrates. (**D**) Blowup of growth cone in (A), line substrate. Red and blue lines mark the locations of two filopodia. Notice back and forth movement of the filopodia and how blue labeled filopodia assembles a robust F-actin cytoskeleton when it aligns. (**E**) Angular trajectories of filopodia that are aligned or not aligned on the ridge patterns. (**F**) Kymograph analysis of filopodial dynamics of aligned filopodia. Bars: (A,D,F) 10 µm.

The most marked differences in morphological responses of neuronal like cells in response to the plain versus the line pattern are observed at the level of the filopodia which have been proposed to work as sensors to guide neuronal growth cones [19]. Thus, we performed high resolution time-lapse microscopy experiments in which we visualized F-actin dynamics using the Lifeact-GFP probe [20], which allows for a high contrast on filopodia (Fig. 4A). On plain substrate, filopodia directly at the growth cone or the neurite shaft extend randomly in multiple directions, perform a typical lateral back and forth motion and then retract. This is accompanied with dynamic neurite protrusion/retraction cycles in multiple directions as described above (Fig. 4A, Movie S3). On the line substrate, we found that the two growth cone filopodia populations displayed different dynamic behaviors (Fig. 4A,B,C, Movies S3 and S4). Filopodia located at the growth cone tip that aligned on the ridges were stable and contained high amounts of F-actin reflected by elevated Lifeact-GFP signal, compared to the non-aligned filopodia (Fig. 4C). Non-aligned filopodia situated on the distal part of the growth cone and throughout the neurite shaft displayed a highly unstable behavior and contained less F-actin (Fig. 4D, Movie S4). To quantitate the dynamics of these different filopodia populations, we tracked their angular evolution. We found that filopodia that are oriented along the lines remained so for hours. In contrast, non-aligned filopodia extend from the neurite shaft with an angle relative to the lines, scan the pattern using a lateral back and forth motion relative to the neurite shaft and then retract, the whole cycle being on the order of five to ten minutes (Fig. 4D, filopodia outlines by red and blue lines, angular motion quantified in Fig. 4E). We also observed that the stochastic search and capture motion performed by these non-aligned filopodia eventually led to their alignement on a ridge of the line substrate. This then subsequently led to the assembly of a robust F-actin cytoskeleton in the newly aligned filopodium (Fig. 4D, filopodia outlined by the blue line). The highly stable extension of aligned filopodia was also apparent with kymograph analyses (Fig. 4F). Occasionally, we also observed some neurites that were not oriented in the direction of the line substrate (Movie S5). These only exhibited unstable filopodia that stochastically scan the pattern through continuous protrusion/retraction cycles coupled with lateral motion, until they finally aligned along a pattern ridge and produced stable, F-actin rich filopodia at the growth cone. These results suggest that filopodia are the organelles that allow sensing of the line substrate through a stochastic filopodia-mediated search and capture mechanism.

**Figure 5:**
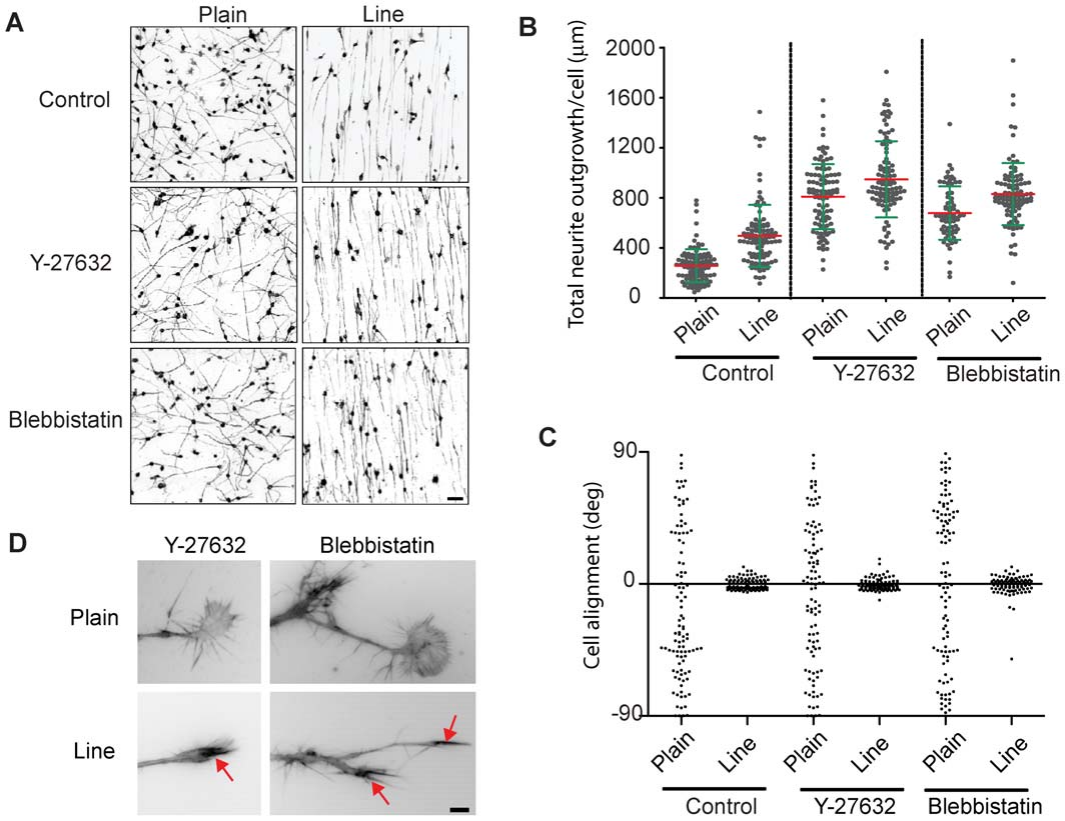
Effect of contractility inhibitors on neurite outgrowth on plain and line substrates. (**A**) Representative images of tubulin-stained N1E-115 cells incubated with 10 µm Y-27632 or 10 µm blebbistatin for 24 h on plain and line substrates. (**B**) Cumulative graph of neurite lengths on a cell basis of the two different treatment and substrate. Mean (red) and standard deviations (green) are shown. Data is based on control of 104 cells for plain and 105 cells for line, Y-27632 treatment of 101 cells for plain, 101 cells for line, blebbistatin of 67 cells for plain and 100 cells for line. P values (T-test) between plain and line substrates <<0.0001. (**C**) Quantification of neurite orientation at 24 hours. Angle measurements of neurite orientation compared to an arbitrary (plain substrate) or to the line direction (line substrate) are shown. (**D**) Growth cone morphologies on plain and line substrates in presence of Y-27632 and blebbistatin drugs. High resolution F-actin pictures are shown. Bars: (A) 100 µm; (D) 5 µm.

Because neuronal guidance in response to immobilized laminin has been reported to require mechanosensing through myosin activation [21], we also explored if contractility is important for neurite orientation in our system through inhibition of Rho kinase or of myosin II ATPase activity (using the Y-27632 and blebbistatin drugs). We observed an increase in neurite length at 24 hours in response to any of the two inhibitors, on both plain and line substrates (Fig. 5A and B). Both drug treatments did not, however, lead to a loss of the ability of the neurite to orient itself on the line substrate (Fig. 5A and C). These drug treatments led to morphological changes of the neurites on the plain substrate in that many neurite tips displayed highly spread, fan-shaped growth cones (Fig. 5D, compare growth cones with those of Fig. 2F), as reported earlier [22], [23]. This was however not observed on the line pattern on which streamlined growth cones with F-actin rich filopodia were still observed. To get insight into the signaling mechanisms that allow the orientation and the steady neurite outgrowth response of the neuronal like cells on the line pattern, we explored if there are global differences in signaling activities in response to ECM topography. For that purpose, we probed lysates of differentiated cells plated on plain or line substrate using western blot analysis for different signaling activities. We performed this experiment at 2 hours when robust initiation of neurite outgrowth is observed, and at 24 hours, when very long but less dynamic neurites occur. We examined post-translational modifications that impact on microtubule stability (microtubule detyrosination and acetylation), MAP kinase signaling (ERK phosphorylation) and adhesion signals (phospho myosin light chain, phospho-FAK and tyrosine-phosphorylated proteins). We could not find any obvious differences in signal intensity between plain and line substrates (Fig. S4). Thus the differences in neurite morphodynamics are most likely dependent on highly localized signaling events involving minute pools of signaling molecules in the growth cone, and therefore might not be resolved with whole cell, global measurements.

## Discussion

**Figure 6:**
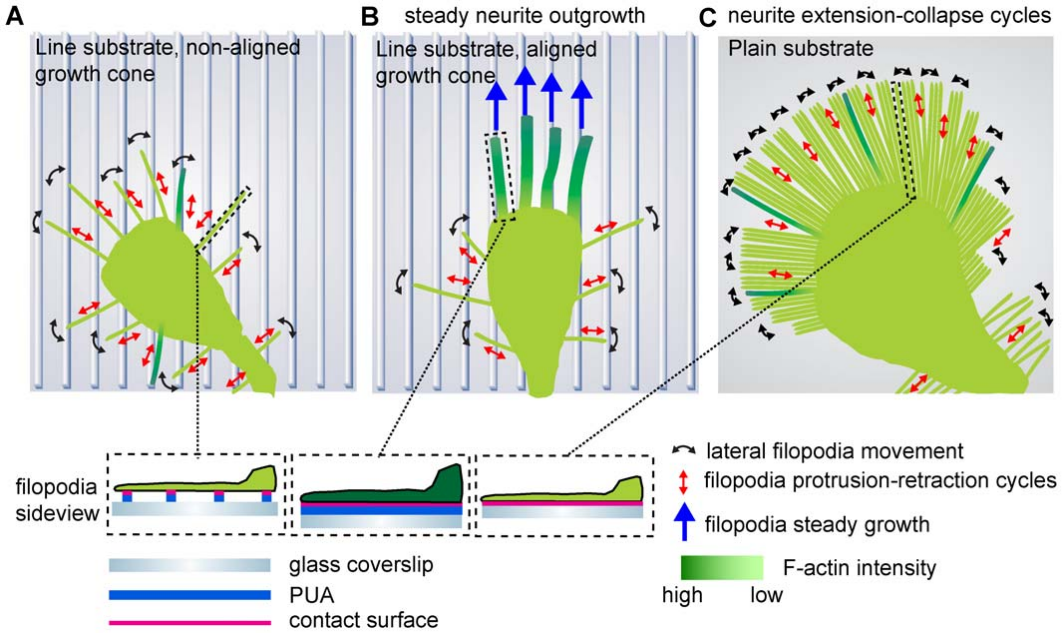
Model of neurite guidance in response to nanotopographical cues. (**A**) Line substrate, unaligned growth cone. Filopodia scan the line substrate through lateral scanning and protrusion/retraction events. Only few filopodia align on the lines and thus almost all filopodia sense only discrete adhesion points to the ECM. (**B**) Line substrate, aligned growth cone. Through stochastic sensing, multiple filopodia have aligned on the line substrate and have assembled an F-actin rich cytoskeleton that stabilizes them. On the distal part of the growth cone, non-aligned, unstable filopodia continue to operate, suggesting a crosstalk between both filopodia populations. This stabilizes the growth cone leading to steady neurite outgrowth. (**C**) Plain substrate. Unrestricted access to ECM leads to a large amount of long filopodia, none of which can be stabilized by a robust F-actin cytoskeleton. This is accompanied with a high frequency of neurite collapse events.

The traditional systems to study neuronal differentiation and the outgrowth of neuronal processes have taken advantage of classic cell culture systems in which ECM proteins are coated homogeneously over 2D surfaces. This is unlikely to recapitulate the neuronal responses occurring *in vivo* in which the ECM is assembled into precise structures which have size features on the nano-meter scale. We find that providing neurons with an anisotropic ECM line substrate, which most likely mimics some topological features of *in vivo* ECM structures, is sufficient to orient neurite outgrowth. Importantly, this is also coupled with a robust increase in neurite outgrowth. Similar responses have already been observed with a variety of different nanometric patterns [9], [24], [25], but a cellular mechanism remained unclear. *In vivo*, similar fibrillar laminin tracks are observed on the surface of Schwann cells, and have been showed to be important for neurite outgrowth and neuronal regeneration after a lesion [4], [5]. Our results that show different neuronal morphologies and morphodynamics in response to the line versus the plain substrate, suggest that oriented neurite outgrowth on the line substrate is the result of a sensing mechanism performed by filopodia. Filopodia are excellent devices to sense the line substrate since they are rigid, rod-like structures that cannot bend and adhere to the grooves of the pattern, and have similar size features than the pattern ridges (filopodia width  = 200–500 nm [26]). They might thus discern if they are aligned or not on the pattern ridges, by evaluating the contact area between the filopodia and the pattern ridges (Fig. 5). Our SEM experiments suggest that non-aligned filopodia only form discrete contacts with ECM molecules on the top of ridges, whereas aligned filopodia can form a more intimate interaction zone with the ridges (Fig. 2G, depicted in Fig. 6A and B). Our time-lapse datasets (Fig. 4) suggest that non-aligned filopodia stochastically scan the line substrate through a process that occurs on a timescale of a couple of minutes, and consists of cycles of protrusion–retraction events that are coupled with a back and forth lateral motion. This is repeated until a filopodium aligns on the pattern ridge which subsequently leads to the assembly of a robust F-actin network and an extensive contact zone with the ridge (Movies S4 and S5, Fig. 6A and B). This then enables to switch off the dynamic unstable behavior observed in non-aligned filopodia, allowing to filopodium stabilization for hours, and ultimately leading to steady neurite outgrowth.

The two distinct filopodial behaviors we observe most likely depend on different levels of coupling between the substrate and the cytoskeleton as proposed in the “molecular clutch model” [27]. Extensive interaction of aligned filopodia with the substrate, might allow a much more efficient cytoskeletal coupling than in non-aligned filopodia, leading to constant filopodial protrusion (e.g. the clutch is continually engaged). In this case, the formation of a robust F-actin network might allow to counteract the actin retrograde flow in the aligned filopodium, leading to its stabilization. In unaligned filopodia, less stable substrate-cytoskeletal coupling might occur, due to the limited interaction with the ECM. In this case, filopodia retraction might occur because strong retrograde flow exceeds actin assembly at the filopodium tip [28] (e.g. the clutch engages and then rapidly disengages). Obviously, in our purely ECM driven system, integrins are the sensors that allow to interprete the line pattern. Consistently, conformationally activated, but unligated integrins have been observed in filopodia of neuronal growth cones [29] and might allow to sense the extent of filopodium contact. One important question is then which signaling events downstream of the integrins allow the formation and maintenance of the robust F-actin network observed in aligned filopodia that allows stabilization of the molecular clutch. A first hint is that this does not occur on the plain substrate, on which each filopodium senses an identical amount of laminin. This suggests that on the line pattern, formation of the robust F-actin network requires integration of spatially regulated adhesive signals from the aligned filopodia at the growth cone tip and from the non-aligned filopodia that are continuously operating on the distal part of the growth cone. However, the signaling events occurring downstream of these receptors remain elusive and our whole-cell measurements of signaling activities certainly could not resolve the precise spatio-temporal regulation of minute pools of signaling molecules in the growth cone that is relevant to this system (Fig. S4). Understanding the signal amplification events that allow the formation of this F-actin rich network will therefore requires advanced live cell imaging techniques that allow to resolve their spatio-temporal dynamics in the growth cone. At the structural level, one can also wonder about the actin binding proteins that allow F-actin stabilization in aligned filopodia? Prime candidates are proteins such as Fascin and Ena/Vasp that enable to crosslink actin filaments into bundles, or myosin-X, a motor protein which seems to be important in localization of filopodial components to the filopodium tip [30].

The specific neuronal guidance mode that we observe on ECM nanotopographic cues is distinct from directional sensing in response to soluble chemo-attractants and –repellants. Rather than the search and capture mechanism, chemotactic growth cone guidance occurs through local stabilization of filopodia most proximal to the attractant source and collapse of those that are distant of the source, leading to net turning in the direction of the chemoattractant [31]. To our knowledge, this has not been shown to involve a robust F-actin network, and illustrates differences between chemotactic and ECM sensing. In vivo, our filopodial search and capture mechanism might therefore allow a basal orientation mechanism along ECM tracks. Additional superposition of gradients of soluble cues might allow to fine tune axonal guidance by inducing growth cone turning at regions such as the midline [32]. Importantly, the filopodia search and capture mechanism that we describe is highly reminiscent of growth cone behavior observed *in vivo*. Live imaging of growth cone dynamics *in vivo* shows similar morphodynamics as for our cells on the line substrate. By example, Xenopus retinal axons display a streamlined growth cone with lateral filopodia that display identical protrusion-retraction behavior coupled with lateral motion than we observe with the non-aligned filopodia on the line pattern [33]. This is accompanied with steady growth without retractions events. Similar growth cone morphologies have also been observed *in vivo* in retinal axons in the mouse [34] or in zebrafish [35]. These different lines of evidence suggest that the precise ECM nanotopology on our line substrate recapitulates geometric features of the *in vivo* ECM.

This raises the issue that the classic 2D substrate does not faithfully reflect the ECM cues that are experienced *in vivo*, as well as the intracellular signaling events that are triggered by the ECM. On classic 2D substrates, unrestricted access to adhesion sites leads to an increase in filopodia length and number on growth cones, neurite shafts and somata. An immediate consequence is that filopodia, owing to their high density and their high adhesive state, cannot perform the highly dynamic behavior of protrusion-retraction coupled with lateral scanning (Fig. 6C). Furthermore they cannot assemble stable, F-actin rich filopodia, most likely because the lack of anisotropy in the ECM that is needed for cell polarization and the production of both filopodia populations. This inability to produce F-actin rich filopodia will then lead to the growth cone collapse events that induce the characteristic protrusion/retraction cycles occurring during neurite outgrowth on the plain substrate. Such protrusion retraction cycles have been documented in multiple neuronal systems, such as by example with stage 2 immature neurites in the classic E18 embryonal hippocampal neurons culture system, just before axonal specification [36]. ECM nanotopology also impacts on the motile behavior of the cell with reduced motility being observed on the line substrate, which also correlates with a low amount of filopodia on the soma. The high degree of motility of neurons observed in classic 2D environments might therefore be a result of the aberrant filopodia formation on the cell soma in response to unrestricted access to adhesion sites that might lead to excessive formation of lamellipodia.

The finding that the sensing mechanism on the line pattern does not require myosin-based contractility highlights different neuronal guidance mechanisms depending on the dimensionality of the laminin ECM. The previously described role of myosin contractility in neuronal guidance stems from experiments in which growth cone turning is evaluated at borders of laminin and poly-ornithine stripes. In such experiments, growth cone turning is inhibited by pharmacological inhibition of myosin [37]. Most likely on such stripes, which have micrometric size features, growth cone filopodia experience the ECM as a 2D environment and use myosin II-based mechanosensing to test rigidity of the surrounding ECM. This might allow them to sense if they are positioned on laminin or not. Interestingly, this mode of neuronal guidance involves exploration of the substrate through neurite extension and retraction cycles [21] as is observed with our cells on the plain substrate. This is in marked contrast with our nanometric line pattern, on which a myosin-independent, filopodia-mediated stochastic search and capture mechanism allows orientation. This allows orientation of neurite outgrowth coupled with steady neurite outgrowth. In this mode of neuronal guidance, growth cone filopodia most likely do not test rigidity by integrin-mediated mechanosensing. Probably, they only measure the differential extent of adhesion surface of aligned and non-aligned filopodia and integrate it in a signaling response that allows the stabilization of aligned filopodia.

To our knowledge, this is the first report that gives insight in how neurons interpret topological cues in the ECM. A clear advantage in our system is that the dynamics of the filopodia mediated search and capture mechanism and of neurite outgrowth are highly stereotypical. This should make it easy to quantify phenotypes in response to perturbation experiments, and thus provides a tractable model system to study neuronal guidance in response to ECM topology. Using the N1E-115 neurite proteome as a template [38], a combination of genetic perturbations and high resolution live cell imaging methods is under investigation to further explore the signaling events that allow to understand how filopodia sense ECM topology and produce steady neurite outgrowth.

## Materials and Methods

### Nanopattern fabrication

The PUA mold was composed of a functionalized prepolymer with acrylate groups for crosslinking, a monomeric modulator, a photoinitiator and a radiation-curable releasing agent for surface activity. The liquid precursor was dropped onto a silicon master that had been prepared by photolithography and a polyethylene terephthalate film was placed on the liquid mixture. For the UV curing, the mold was exposed to UV (λ = 250–400 nm) light for 25 seconds and peeled off from the master. The 18-mm-diameter glass cover slip was treated by oxygen plasma for 1 minute and then coated with glass primer by spin coating at 4000 rpm for 30 seconds. After rinsing with isopropyl alcohol for 3 min, glass coverslip was dried using N_2_ gas. For the nanopattern fabrication, PUA liquid was dropped onto a prepared glass substrate and the PUA mold was attached on the PUA droplet. The PUA droplet was cured by UV exposure for 25 seconds and the mold was peeled off from the glass substrate.

### Cell culture

N1E-115 neuroblastoma cells (American Tissue Culture Collection) were cultured in Dulbecco's modified Eagle's medium (DMEM) (Invitrogen) supplemented with 10% FBS, 1% Glutamin, and 1% penicillin/streptomycin. For differentiation, N1E-115 cells were starved for 24 h in serum-free neurobasal medium (Invitrogen) supplemented with 1% Glutamin, and 1% penicillin/streptomycin. Cells were then detached with PUCK's saline, and an adequate number of cells were replated on coverslips (plain substrate) or coverslips with the PUA pattern (line substrate). These coverslips were previously coated with 10 ug/ml laminin for 2 h at 37°C. For experiments with primary cells, cortical neurons were isolated.

### Immunofluorescence, transfection and time-lapse imaging

For immunofluorescence, cells were quickly washed in PBS, fixed in BRB80 (80 mM PIPES, 1 mM MgCl2, 1 mM EGTA, pH 6.8) containing 0.25% glutaraldehyde for 30 seconds and permeabilized in BRB containing 0.1% Triton X-100, 0.25% glutaraldehyde in BRB80 for 10 minutes. Coverslips were then washed with PBS, incubated in freshly prepared 0.2% sodium borohydride in PBS for 20 min, followed by two additional PBS washes. Coverslips were then blocked in antibody solution (2% BSA, 0.1% Triton-X100 in PBS) for 10 min. For staining, cells were incubated with alexa-fluor 488 labeled phalloidin (Invitrogen), an anti α-tubulin antibody (Sigma), an Alexa-fluor 546 conjugated secondary antibody, and DAPI for 30 min. After 3 PBS washes, cells were mounted on a coverslip using Prolong gold (Invitrogen) as mounting medium. All microscopy was performed using an Eclipse Ti microscope (Nikon) steered by Metamorph software (Molecular Devices) and using a LED-based light source (Coolled). Low resolution images of neurons on coverslips, used to measure neurite length were acquired using Nikon fluorescence microscope using a 10x air objective. For neurite outgrowth analysis, about 25 fields of view were automatically captured using a motorized stage. Different measurements of fluorescence intensities were performed using metamorph. High resolution actin and tubulin images were acquired using an oil-immersion 60x high NA objective. For transfection, 3×10^5^ N1E-115 cells were plated in one dish of a 6-well plate. The next day cells were transfected with 2 µg of plenti Lifeact-GFP using 4 µl of FuGENE transfection reagent (Roche) per well in presence of serum. 24 hours post-transfection cells were starved in neurobasal medium, and 48 hours post-transfection cells were replated on coverslips. For time-lapse imaging, 2×10^4^/well were replated in 12-well plate previously coated with 10 µg/ml laminin and allowed to attach for three hours. The glass coverslip was transferred to a Ludin Chamber (Life imaging services) and filled with neurobasal medium supplemented with 20 mM Hepes. Multi-stage time-lapse imaging was then performed.

### Image and statistical analysis

All image analysis was performed using metamorph software (Universal Imaging). For neurite outgrowth analysis (Fig. 1E), average cell neurite length was measured on the multiple images using the neurite outgrowth plugin. To measure F-actin intensities in filopodia (Fig. 2E and 4C), mean fluorescence intensity of a region of interest was computed and normalized to surface. This was then further normalized to the lower F-actin intensities. Cell alignment (Fig. 1D) and filopodium angle measurements (Fig. 4C and E) were performed manually using the line scan tool. Tracking of neurite tip (Fig. 3B) was performed manually using the track points plugin. Statistical analysis was performed using two-tailed t-test except for total neurite outgrowth analysis, for which one-way ANOVA test was used to determine statistical differences between plain and the line substrates with different spacings. A value of p<0.05 is considered statistically significant.

## Supporting Information

Figure S1 **Laminin coating efficiency and neurite outgrowth analysis. (A)** Confocal microscope image of PUA pattern autofluorescence (405 nm laser) and Alexa 561-labeled laminin (563 nm laser). Scheme shows at which Z positions confocal images were focused. **(B)** Examples of tubulin and DAPI images used for Metamorph neurite outgrowth analysis. Red signal in Metamorph image analysis panels show fidelity of image segmentation. In the segmentation image, each cell with its neurites are color-coded specifically. Bars: (A) 200 nm, (B) 200 µm.
(TIF)

Figure S2 **Behavior of primary cortex neurons on plain and line substrates.** Freshly isolated cells were plated on glass coverslips coated with 100 ug/ml poly-D-ornithine and 10 ug/ml laminin and allowed to extend neurites for 5 days. Coverslips were then fixed and stained for DAPI and Tuj-1 b3-tubulin antibodies. Right panels show blowups from the inset in the line substrate images. Fluorescence intensities in these images have been rescaled to show the line pattern.
(TIF)

Figure S3 **Cell soma motility. (A)** Phase contrast time-lapse series of cells on plain and line substrates. Colored circles indicate cell soma positions. **(B)** Overlay of cell soma positions from time-lapse series on plain and line substrate. Quantification of cell body instantaneous speed on plain and line substrate. P values (T-test) << 0.0001 in both cases. Bars: (A,B,C) 25 µm.
(TIF)

Figure S4 **Signaling activities of N1E-115 cell populations on plain and line substrates. (A)** Relative protein enrichment in plain (P) and line (L) for 2 h and 24 h was analyzed by western blot. **(B)** Phase contrast micrographs of N1E-115 cells used for western blot analysis. Bar: (A) 100 µm.
(TIF)

Movie S1 Phase contrast timelapse series of N1E-115 on the plain and line substrates as shown in Fig. 4. Movie started 2 hours post-plating. Note the processive neurite outgrowth behavior of cells on the line substrate. Time is in hours:minutes. Bar: 50 µm.
(MP4)

Movie S2 Phase contrast timelapse series of N1E-115 on the plain and line substrates as shown in Fig.S2. Note the highly motile behavior of the cell on the plain substrate. Time is in hours:minutes. Bar: 50 µm.
(MP4)

Movie S3 Fluorescence timelapse serie of lifeact-GFP transfected N1E-115 growth cones on the plain and line substrates. Note the highly processive neurite outgrowth behavior on the line pattern, as well as the F-actin rich filopodia at the growth cone. Time is in minutes:seconds. Bar: 10 µm.
(MP4)

Movie S4 Fluorescence timelapse serie of lifeact-GFP transfected N1E-115 growth cones on the line substrate. Note the distinct morphodynamic behaviors of the two different filopodia populations. Time is in minutes:seconds. Bar: 10 µm.
(MP4)

Movie S5 Fluorescence timelapse serie of lifeact-GFP transfected N1E-115 cell on the line substrate. Time is in minutes:seconds. Note how F-actin rich filopodia can form when the neurite aligns along the lines at the end of the movies. Bar: 10 µm.
(MP4)
