# Supplementary figures and images for: Two Distinct Filopodia Populations at the Growth Cone Allow to Sense Nanotopographical Extracellular Matrix Cues to Guide Neurite Outgrowth
Source: PLoS One. 2010 Dec 30;5(12):e15966. doi: 10.1371/journal.pone.0015966 (PMC3012734; doi:10.1371/journal.pone.0015966)

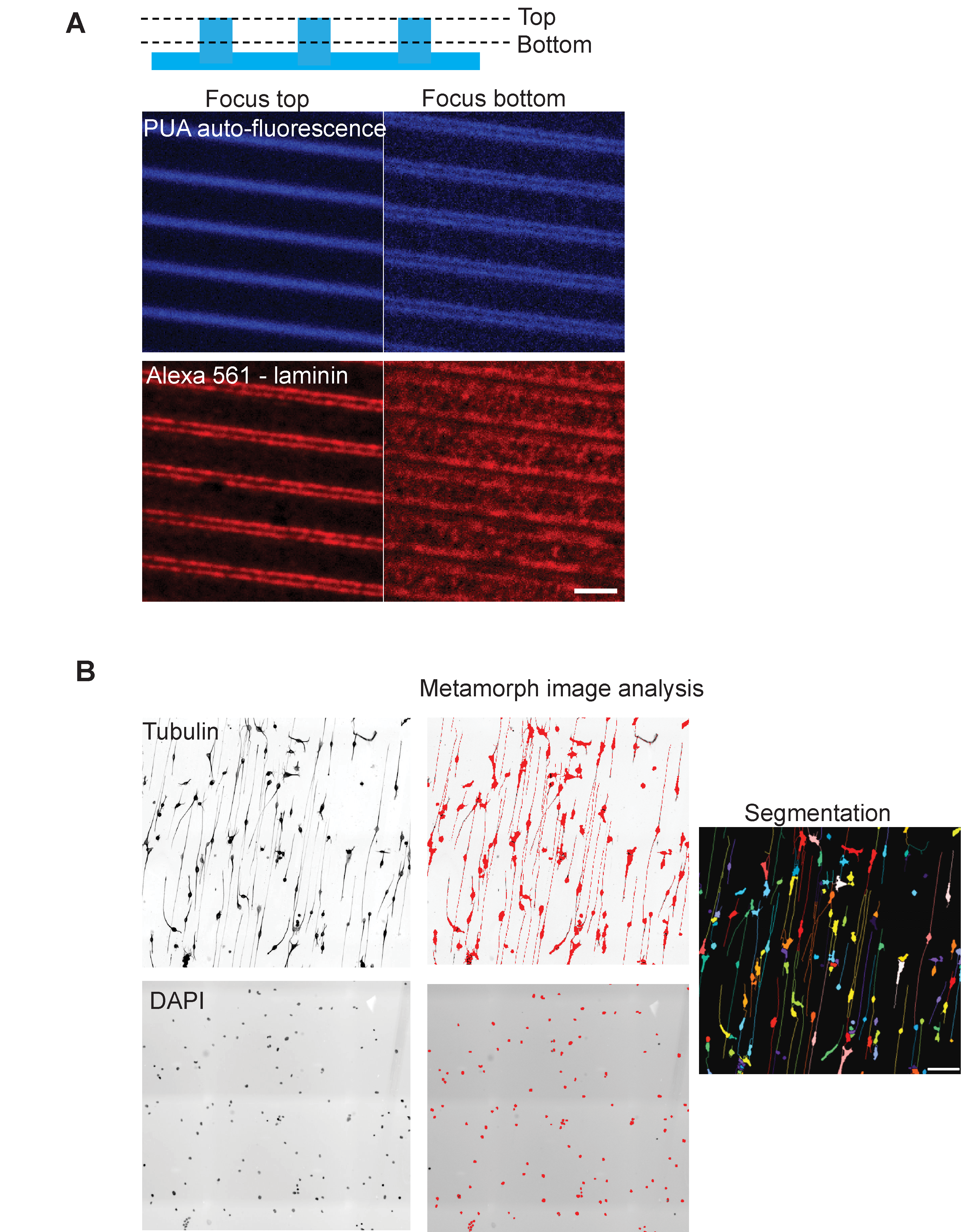

Supplement: Figure S1 — Laminin coating efficiency and neurite outgrowth analysis. (A) Confocal microscope image of PUA pattern autofluorescence (405 nm laser) and Alexa 561-labeled laminin (563 nm laser). Scheme shows at which Z positions confocal images were focused. (B) Examples of tubulin and DAPI images used for Metamorph neurite outgrowth analysis. Red signal in Metamorph image analysis panels show fidelity of image segmentation. In the segmentation image, each cell with its neurites are color-coded specifically. Bars: (A) 200 nm, (B) 200 µm. (TIF) [file pone.0015966.s001.tif]

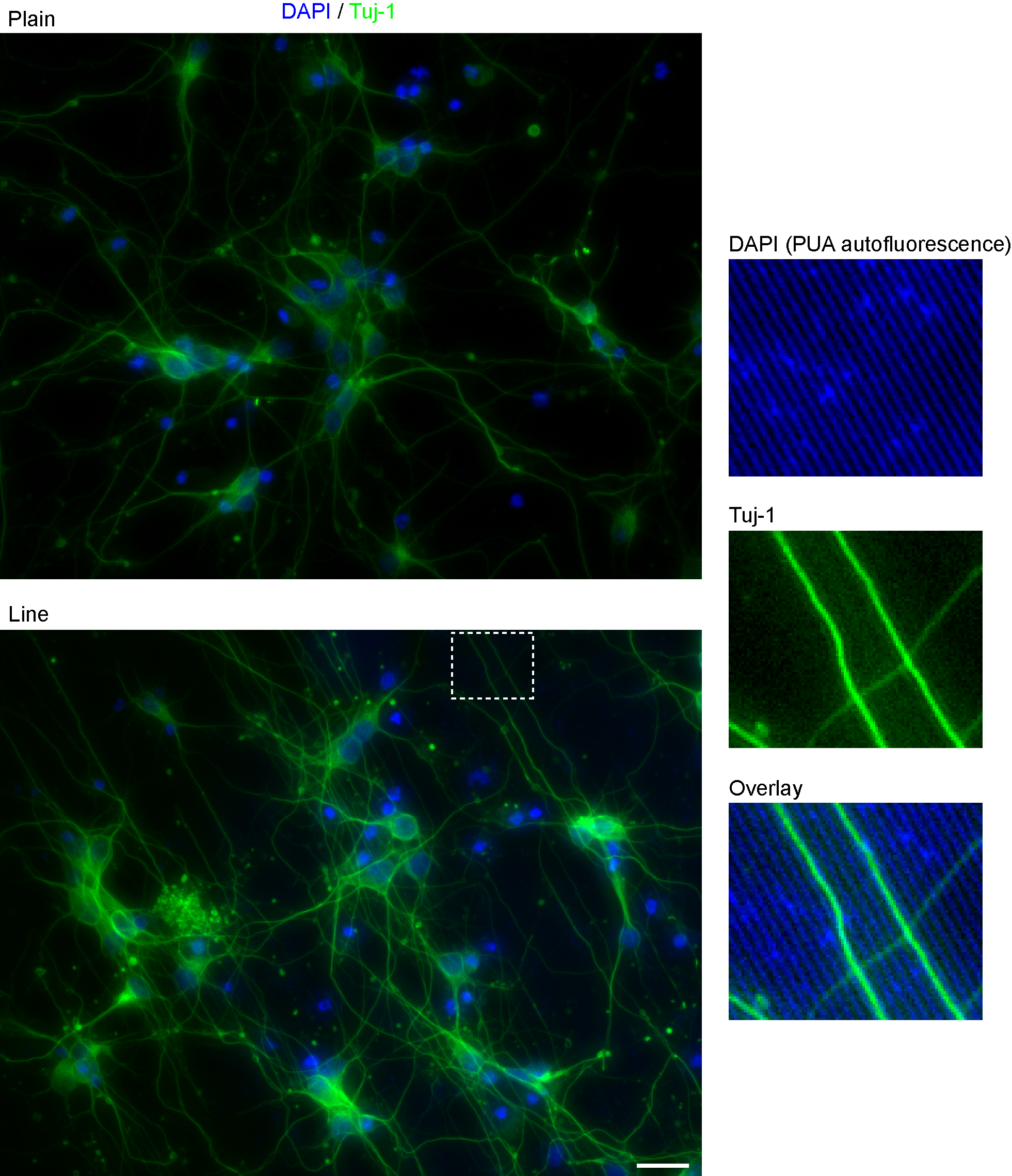

Supplement: Figure S2 — Behavior of primary cortex neurons on plain and line substrates. Freshly isolated cells were plated on glass coverslips coated with 100 ug/ml poly-D-ornithine and 10 ug/ml laminin and allowed to extend neurites for 5 days. Coverslips were then fixed and stained for DAPI and Tuj-1 b3-tubulin antibodies. Right panels show blowups from the inset in the line substrate images. Fluorescence intensities in these images have been rescaled to show the line pattern. (TIF) [file pone.0015966.s002.tif]

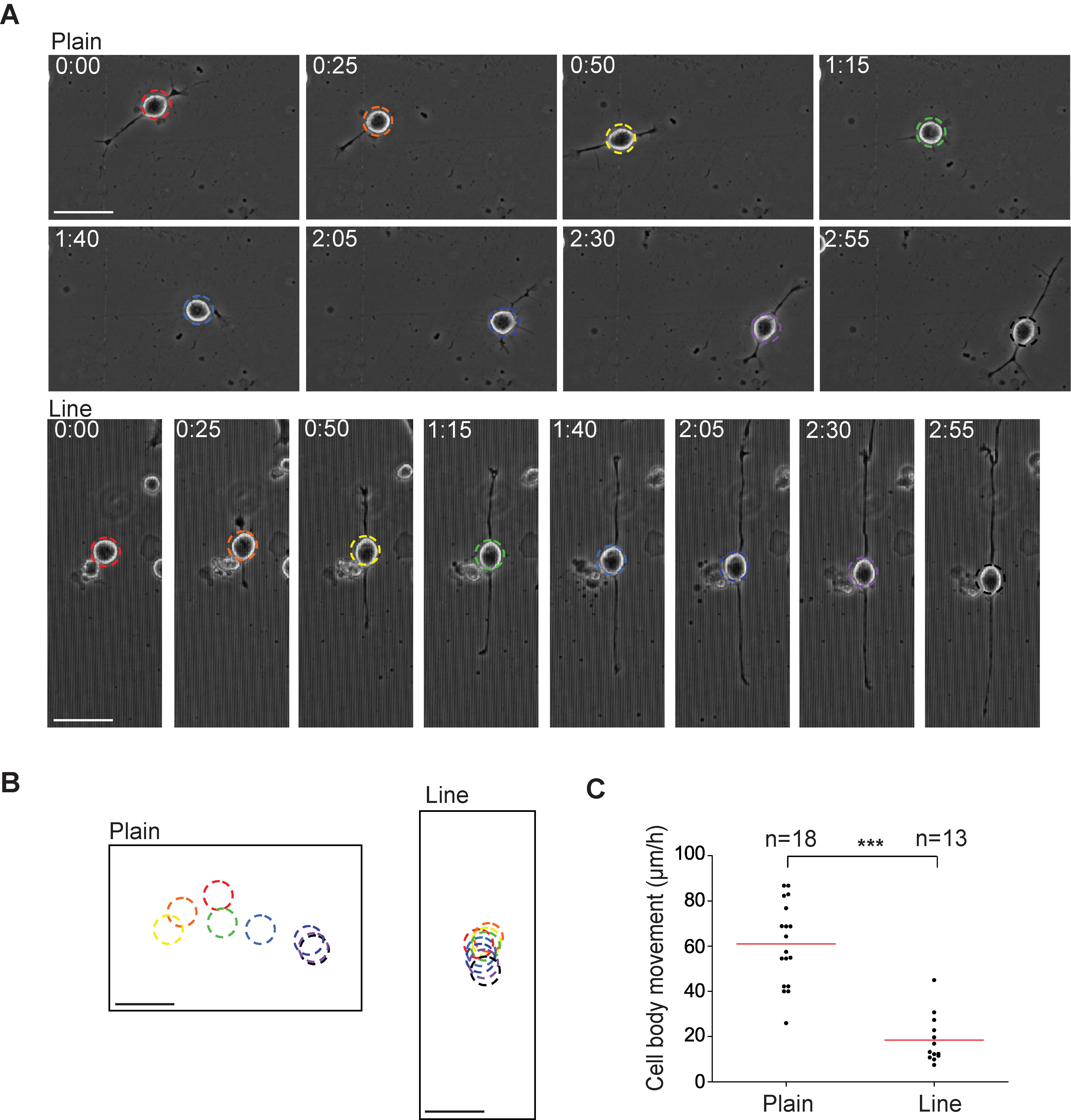

Supplement: Figure S3 — Cell soma motility. (A) Phase contrast time-lapse series of cells on plain and line substrates. Colored circles indicate cell soma positions. (B) Overlay of cell soma positions from time-lapse series on plain and line substrate. Quantification of cell body instantaneous speed on plain and line substrate. P values (T-test) << 0.0001 in both cases. Bars: (A,B,C) 25 µm. (TIF) [file pone.0015966.s003.tif]

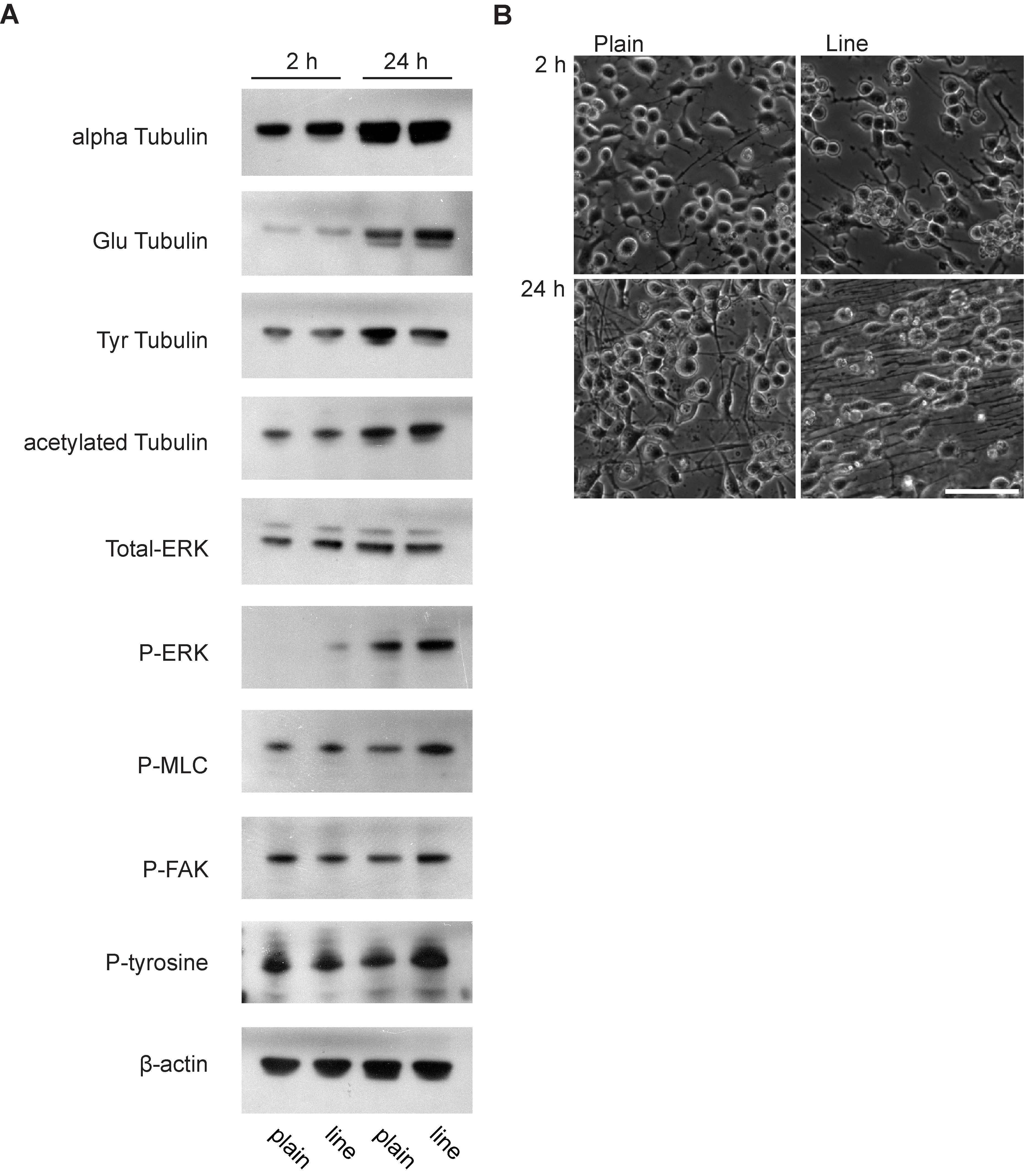

Supplement: Figure S4 — Signaling activities of N1E-115 cell populations on plain and line substrates. (A) Relative protein enrichment in plain (P) and line (L) for 2 h and 24 h was analyzed by western blot. (B) Phase contrast micrographs of N1E-115 cells used for western blot analysis. Bar: (A) 100 µm. (TIF) [file pone.0015966.s004.tif]
